# A versatile GPCR toolkit to track in vivo neuromodulation: not a one-size-fits-all sensor

**DOI:** 10.1038/s41386-021-00982-y

**Published:** 2021-02-18

**Authors:** Marie A. Labouesse, Tommaso Patriarchi

**Affiliations:** 1 Department of Psychiatry, College of Physicians and Surgeons, Columbia University, Columbia, NY USA; 2 Division of Molecular Therapeutics, New York State Psychiatric Institute, New York, NY USA; 3 Institute of Pharmacology and Toxicology, University of Zurich, Zurich, Switzerland; 4 Neuroscience Center Zurich, University and ETH Zurich, Zurich, Switzerland

**Keywords:** Microscopy, Neurophysiology

## A versatile toolkit of GPCR-sensors to track neuromodulation

**Fig. 1:**
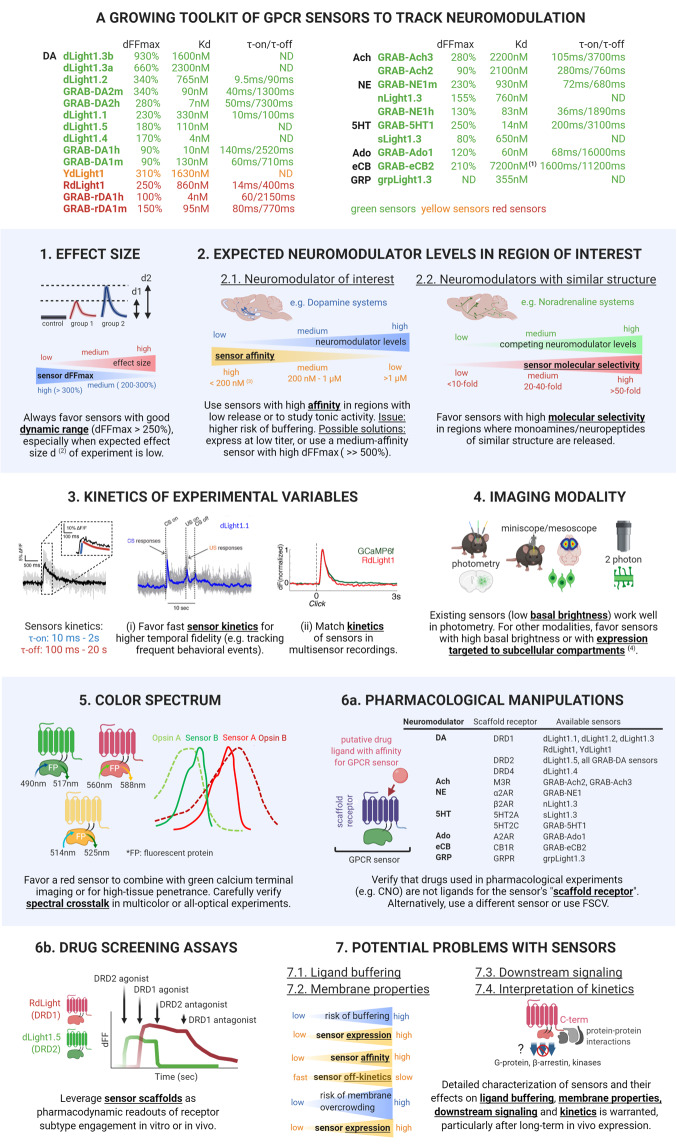
Choosing a neuromodulator GPCR sensor, a balancing act (see main text for details). (1) *K*_d_ for 2AG (reported *K*_d_ for AEA: 500 nM [10]). (2) Effect size (*d*1, *d*2): magnitude of change in neuromodulator levels between groups, estimated e.g. by calculating Cohen’s *d*: delta of the means of the groups, normalized to the pooled standard deviation [19]. (3) ”High” and “low” affinity denominations are relative (here chosen based on DA/NE systems) and may be shifted for other neuromodulators. (4) Expected future developments. 5HT serotonin, Ach acetylcholine, Ado adenosine, CNO clozapine-N-oxide, C-term C-terminal, DA dopamine, DRD1/DRD2 DA receptor 1 and 2, dFFmax dynamic range (maximal dFF), eCB endocannabinoid, FP fluorescent protein, FSCV fast-scan cyclic voltammetry, GRP gastrin-like peptide, *K*_d_ apparent affinity, ND not determined, NE noradrenaline, τ-on/τ-off on- and off-sensor kinetics (half-rise, decay times).

Measuring the real-time dynamics of neuromodulator release in the brain with subcellular resolution is a long-sought goal in neuroscience, due to the immense implications for basic science and medicine. The past 3 years have brought this goal within reach, with the appearance of a new class of genetically encoded fluorescent sensors for neuromodulators [1–11] (Fig. 1) constructed using G-protein-coupled-receptors (GPCR) [12]. GPCR sensor design takes advantage of the fact that most neuromodulators harbor GPCRs as their native receptors, and builds on protein engineering expertise acquired through work on genetically encoded calcium sensors [13–16]. High-throughput screening techniques are used to incorporate circularly permuted fluorescent proteins (cpFP) within GPCRs of interest, enabling the optical visualization of neuromodulator dynamics [17]. Given the diversity of naturally existing GPCR scaffolds, there is a large realm of opportunities to generate new GPCR-sensors with tailored properties adapted for each neuromodulator. The dLight1 family exemplifies this possibility, providing a panoply of eight sensors engineered using DRD1, DRD2, and DRD4 receptor subtypes, each with different properties [18]. The rapid developments in GPCR sensor engineering are now allowing an ever-growing ability to tailor sensor use to specific experimental applications, but may create a dilemma for end-users pondering which sensor is best suited for their work or how to interpret results.

## To the neuroscientist end-user: sensor choice recipe in 6 key steps

Here we provide a step-by-step recipe for end-users (Fig. 1, Supplementary Table S1) to guide sensor choice:

### Expected effect size of the experimental manipulation

A sensor’s dynamic range (dFF_max_) (~50% to ~1000%) provides an estimate of the range of responses that can be obtained against varying neuromodulator concentrations. Sensors with good dFF_max_ (which we consider >250%) are always preferable, but particularly when the experiment’s effect size (=magnitude of changes in neuromodulator levels, normalized to the standard deviation [19]) is expected to be low (e.g. when measuring changes in tonic release, release in regions with low neuromodulator innervation, or to identify small, dose–response changes in release).

### Expected neuromodulator levels in brain region of interest

Existing GPCR-sensors harbor apparent affinities (*K*_d_) ranging from 4 nM up to 7 µM, providing a broad range of detection windows that should be matched to the expected local neuromodulator levels. Indeed, the affinity-based model for receptor–ligand (R–L) interactions [20] posits that, at equilibrium, the fractional occupancy of receptors *f* depends on the ligand concentration [L] and the receptor’s apparent affinity *K*_d_: $f = [{\mathrm{L}}]/({K}_{{\mathrm{d}}} + [{\mathrm{L}}])$. Extending this model to GPCR-sensors, one can predict that sensors should work best when half of the sensors are occupied (*f* = 50%), i.e. when ligand concentrations [L] are close to the *K*_d_. For example, a medium affinity sensor for DA (e.g. *K*_d_ = 500 nM) is poorly occupied (*f* = 4%) at [DA] = 20 nM and thus may not reliably detect tonic DA changes or phasic DA release in regions poorly innervated by DA projections, but should work well in the 100–1000 nM window (~phasic DA in striatum [21, 22]), and only saturate at high (micromolar) concentrations. High-affinity variants (<200 nM) are likely well-suited for capturing tonic release or for regions with low DA innervation (e.g. cortex) but have an elevated risk for ligand buffering (see below). Medium affinity variants with excellent dynamic range (dFF_max_ ≫ 500%) may represent a good alternative to increase the breadth of the detection range (higher dFF change for the same change in sensor occupancy). Importantly, in brain regions where multiple neuromodulators of similar structure are released, it is essential to favor sensors with high molecular selectivity, e.g. when tracking DA over NE (*K*_d-DA_ ≫ *K*_d-NE_) [11].

### Kinetics of the experimental variables

Sensor on/off-kinetics are highly variable (τ-on: 10 ms–2 s; τ-off: 100 ms–20 s) and should be interpreted with caution (see below). Fast kinetics are generally preferred to track endogenous release dynamics as closely as possible, especially when high temporal resolution is required, e.g. to track the response to closely related events (e.g. cues, optogenetic stimulation) or rapid changes in behavior. In particular, fast on-kinetics will increase sensor responses to brief release events, boosting sensitivity. Fast off-kinetics will also reduce the chance of ligand buffering (τ-off inversely proportional to the dissociation rate constant *k*_off_ [22]). Slow off-kinetics on the other hand, will integrate temporally close release events resulting in a large global response at the cost of temporal detection accuracy, akin to GCaMP6s/7 s (“slow”). Fast kinetics are less critical when tracking average release across hours/days, or when measuring the release of certain slow-acting (minutes) neuropeptides, e.g. gastrin-like peptide [11].

### Imaging modality

Existing GPCR-sensors are optimized for fiber photometry given their low basal brightness and relatively high evoked fluorescence. Future sensor variants with higher basal brightness, improved evoked fluorescence and/or subcellular targeting to individual compartments (e.g. soma vs. dendrites) will be necessary to allow identification and tracking cellular or subcellular release events under miniscope or 2-photon modalities.

### Multicolor experiments

GPCR-sensors are compatible with 2-color imaging (e.g. with calcium indicators [1, 2, 4, 10]). If available, red neuromodulator sensors [2, 4] are advantageous in combination with green axon terminal calcium imaging (red terminal imaging is very sensitive to bleaching). Red sensors also offer higher tissue penetrance; which should produce higher SNR in photometry or increase 2-photon maximal imaging depth. GPCR-sensors can also be implemented in all-optical experiments using optogenetic manipulations [1, 2, 4, 7, 10] at orthogonal wavelengths (see refs. [1, 23]). There is a distinct risk for spectral crosstalk between opsin and sensor which should always be tested for by using appropriate controls (see ref. [24]).

### Pharmacological considerations

GPCR-sensors are engineered using human neuromodulator receptors and thus can respond to pharmacological ligands. This provides a useful tool to validate neuromodulator sensors using antagonists (see refs. [1, 23]), but also means GPCR-sensors are incompatible with certain pharmacological manipulations. In such cases, sensors responsive to the drug of interest should be avoided in favor of sensors built on a different receptor subtype, or alternatively one can use periplasmic-binding-protein (PBP) sensors [25] or fast-scan cyclic voltammetry (FSCV).

## To the drug hunter: a future with receptor-subtype sensor families for drug screening?

The pharmacological characteristics of GPCR-sensors also represent a unique opportunity for novel drug discovery assays using multicolor fluorescent technology. We provided proof of concept for this possibility by screening DRD1 and DRD2 ligands against red DRD1 and green DRD2 sensors in vitro [2]. Such assays could presumably be deployed in vivo [1–5, 7] to probe pharmacodynamic target engagement of specific receptor subtypes during behavior following drug administration, with high spatiotemporal resolution and cell specificity.

## A cautionary tale: potential problems with GPCR-sensors

GPCR-sensors have several limitations that end-users should be aware of.

### Ligand buffering?

There is a risk for sensors to buffer endogenous ligands, i.e. reducing neuromodulator availability at native receptors and in turn affecting endogenous downstream signaling. We verified in vitro [1] that sensor expression does not affect neuromodulator-induced cAMP signaling. However, whether long-term expression of sensors in the intact brain induces ligand buffering is unknown. To address this, one could measure the impact of sensor expression (at increasing concentrations, i.e. increasing AAV titers) on native neuromodulator dynamics obtained with FSCV or other functional (e.g. PKA or cAMP [26, 27]), physiological (e.g. cell firing properties), neuroanatomical (e.g. inflammatory markers) or behavioral readouts. Mathematical modeling could help estimating the risk of ligand buffering, for example by calculating the quantity [LS] of ligand molecules bound to a sensor (sensor affinity *Kd*_S_) and comparing it to the quantity [LR] of ligand molecules bound to a native receptor (receptor affinity *Kd*_R_). We could then use affinity-based models for receptor–ligand interactions [20, 22] which posit that, for two independent receptor populations S and R of concentrations *B*max_S_ and *B*max_R_, specific binding = [LS] + [LR] at equilibrium. Ligand buffering at sensors would equate: [LS] = *B*max_S_ × [L]/(*Kd*_S_ + [L]) and ligand binding at native receptors: [LR] = *B*max_R_ × [L]/*Kd*_R_ + [L]). Although this model has its limitations [22], it can make several predictions: (i) The risk of ligand buffering [LS] increases with sensor concentration *B*max_S_. At present, the concentration of sensors has not been determined (see next paragraph), and it is therefore not possible to determine whether ligand buffering is a significant phenomenon or not. (ii) The risk of ligand buffering increases inversely with the sensor’s *Kd*_S_. Thus, high-affinity sensors (low *Kd*_S_ values) should be expressed at concentrations (*B*max_S_) as low as possible to ensure low risk of buffering: [LS] ≪ [LR]. Sensors with slow off-kinetics (high τ-off) also increase the risk of buffering since *Kd*_S_ is inversely proportional to τ-off [20]. (iii) The impact of ligand buffering on native receptor function will depend on their affinities *Kd*_R_. For example, the reported affinity of DA receptors DRD1 and DRD2 are *Kd*_DRD1_ = 1600 nM and *Kd*_DRD2_ = 25 nM, respectively [28]. In the condition when concentrations of sensors *B*max_S_ and receptors *B*max_R_ are equal (=*B*max), at low DA concentrations ([DA] = 20 nM), a high affinity sensor (*Kd*_S_ = 50 nM) has a high chance of affecting DA binding at DRD2 since binding would be of similar magnitude at the sensor and at the DRD2 receptor: $\left[ {{\mathrm{{DA}}} \,-\, S} \right] \,=\, 29\% \,\times\, B{\mathrm{{max}}} \,\approx\, \left[ {{\mathrm{{DA}}} \,-\, {\mathrm{{DRD2}}}} \right] = 45\% \times B{\mathrm{{max}}}$. Since DRD1 is less sensitive to low basal DA (low binding at DRD1: $\left[ {{\mathrm{{DA}}} - {\mathrm{{DRD}}}1} \right] = 1.2\% \times B{\mathrm{{max}}}$), such high-affinity sensors are less likely to have a buffering effect on this receptor subtype at low [DA]. Upon phasic DA release ([DA] = 200 nM), the same high-affinity sensor (*Kd*_S_ = 50 nM) will have a lesser effect on DRD2 (DRD2 close to saturation: $\left[ {{\mathrm{{DA}}} - {\mathrm{{DRD}}}2} \right] = 89\% \times B{\mathrm{{max}}}$) but could strongly impact binding at DRD1 ($\left[ {{\mathrm{{DA}}} - S} \right] = 80\% \times B{\mathrm{{max}}}{\kern 1pt}\,{\mathrm{{vs.}}}\, \left[ {{\mathrm{{DA}}} - {\mathrm{{DRD}}}1} \right] \gg 11\% \times B{\mathrm{{max}}}$). This illustrates how sensors also need to be carefully chosen based on whether changes in tonic DA release (e.g. DA dips at DRD2 [26]) or phasic release are under study. Of course, it must be noted that (i) the spatiotemporal dynamics of release and reuptake [21, 29] and (ii) the number of sensors expressed near the sites of release and exposed to the neuromodulator will further dictate the kinetics and significance of ligand buffering and would need to be incorporated into mathematical models.

### Membrane overcrowding?

GPCR-sensors are expressed at the membrane but lack ligand-induced internalization. Although their turnover is not fully understood, it is possible that their surface levels increase over time, which could lead to membrane overcrowding, and in turn affect membrane properties. Acute in vitro dLight1 expression was estimated ~10-fold higher relative to endogenous GPCRs [30] and this expression level did not affect endogenous GPCR signaling pathways [1]. However, neither the level of sensors expressed in vivo, nor the impact on in vivo membrane physiology (e.g. excitability, oligomerization) or toxicity (e.g. cell death) are known and should be addressed in future work. Sensor concentrations in tissue obtained using increasing AAV titers could be quantified using classical radioligand-binding assays; one could expect values around ~1 pmol/mg protein as shown for striatal transgenic DRD2 [31]. High-resolution estimates of sensor expression in functional compartments (e.g. dendrites) obtained using fluorescent tags [32] could also be useful. This would allow to estimate the quantity of sensors actually trafficked near sites of release and thus susceptible to contribute to (1) the fluorescent signal, (2) membrane overcrowding, and (3) ligand buffering.

### Impact on downstream signaling?

Since GPCR are membrane receptors, they interact with cellular proteins to induce downstream signaling. It was verified that GPCR-sensors do not couple with G-protein or beta-arrestin pathways [1, 3]. However, GPCRs, in particular their C-terminus, are involved in a multitude of other protein–protein interactions, including kinases (e.g. PKA/PKC, GRK) and other scaffold proteins (e.g. PDZ-domain-containing proteins) [33] which should be investigated in future work.

### Interpretation of transient kinetics?

Kinetics of obtained data should be interpreted with caution [25]. Ideally, sensor on/off-kinetics primarily reflect the kinetics of exposure (release/clearance) to the neuromodulator. However, they are likely also influenced by the sensor’s structure, which impacts kinetics of ligand binding/unbinding and conformational dynamics. For example, since GPCR-sensors do not couple with G-proteins, they likely cannot adopt the “high-affinity” orthosteric state induced by G-protein binding [34]. This may affect ligand binding/unbinding dynamics observed at GPCR-sensors and in turn impact (1) the kinetics of the sensor and therefore also (2) the kinetics of measured transients.

## Funding and disclosure

This work was made possible by funding from the European Research Council (ERC) under the European Union’s Horizon 2020 research and innovation program (Grant agreement Nos. 891959 and 101016787) to TP, and Swiss National Science Foundation grants (#P400PB_180841, #P4P4PB_191069) to MAL. We also acknowledge funding from Swiss National Science Foundation (Grant No. 310030_196455), Novartis Foundation for Medical–Biological Research, Olga Mayenfisch Foundation, Hartmann Müller Foundation for Medical Research grants to TP; TP is listed as inventor on a patent application related to the technology described in this article. MAL has nothing to disclose.

## Supplementary information

Supplementary Table 1
