# Supplementary material for: A versatile GPCR toolkit to track in vivo neuromodulation: not a one-size-fits-all sensor
Source: Neuropsychopharmacology. 2021 Feb 18;46(12):2043–7. doi: 10.1038/s41386-021-00982-y (PMC8505436; doi:10.1038/s41386-021-00982-y)
Supplement: Supplementary file 1 — Supplementary Table 1 [file 41386_2021_982_MOESM1_ESM.pdf]

Supplementary Table S1

A versatile GPCR toolkit to track in vivo neuromodulation: not a one-size-fits-all sensor

Marie A. Labouesse (PhD) [1,2,3], Tommaso Patriarchi (PhD) [3,4,*]

[1] Department of Psychiatry, College of Physicians and Surgeons, Columbia University, NY, USA
[2] Division of Molecular Therapeutics, New York State Psychiatric Institute, NY, USA
[3] Institute of Pharmacology and Toxicology, University of Zurich, Zurich, Switzerland
[4] Neuroscience Center Zurich, Zurich, Switzerland
***** Correspondence: Prof. Dr. Tommaso Patriarchi, Institute of Pharmacology and Toxicology, Irchel Campus Y17, University of Zurich, Winterthurerstrasse 190, CH-8057 Zurich. Tel.: (+41) 044 635 59 21. Email: patriarchi@pharma.uzh.ch

Neuro-modulator	Sensor (green, unless noted otherwise)	GPCR	1-photon Exc./Emis.	Dynamic range (dFF_{max})	Affinity (K_d/EC_{50})	Molecular specificity (DA vs. NE)	$t_{1/2}$ rise time (σ_{on})	$t_{1/2}$ decay time (σ_{off})	Imaging in vitro/ex vivo	Imaging modalities in vivo in rodents	Model systems in vivo	Source
DA	dLight1.1	DRD1	490/517 nm	230 % *	330 nM *	70-fold(NE)	10 ms ***	100 ms ***	in vitro [1]	photometry, miniscope, 2-photon (non-cellular ROIs) in mice [1]	mouse, rat	[1]
	dLight1.2	DRD1	490/517 nm	340 % *	765 nM *	ND	9.5 ms ***	90 ms ***	in vitro, ex vivo [1]	photometry [2], 2-photon (non-cellular ROIs) [1]	mouse	[1]
	dLight1.3a	DRD1	490/517 nm	660 % *	2300 nM *	ND	ND	ND	in vitro [1]	ND	ND	[1]
	dLight1.3b	DRD1	490/517 nm	930 % *	1600 nM *	270-fold(NE)	ND	ND	in vitro [1,3]	photometry [3]	mouse, rat, drosophila	[1,3]
	dLight1.4	DRD4	490/517 nm	170 % *	4 nM *	ND	ND	ND	in vitro [1]	ND	ND	[1]
	dLight1.5	DRD2	490/517 nm	180 % *	110 nM *	ND	ND	ND	in vitro [1,4]	ND	ND	[1]
	RdLight1 (red)	DRD1	560/588 nm	250 % *	860 nM *	60-fold(NE)	14 ms ***	400 ms ***	in vitro, ex vivo [4]	photometry [4]	rat	[4]
	YdLight1 (yellow)	DRD1	514/525 nm	310 *	1630 nM *	ND	ND	ND	in vitro [4]	ND	ND	[4]
	GRAB-DA1m	DRD2	490/510 nm	90 % *	130 nM *	10-fold(NE)	60 ms *	710 ms *	in vitro, ex vivo [5]	photometry [5]	mouse, drosophila, zebrafish	[5]
	GRAB-DA1h	DRD2	490/510 nm	90 % *	10 nM *	10-fold(NE)	140 ms *	2520 ms *	in vitro, ex vivo [5]	photometry [5]	mouse	[5]
	GRAB-DA2m	DRD2	500/520 nm	340 % *	90 nM *	22-fold(NE)	40 ms *<br>140 ms ****	1300 ms *<br>8200 ms ****	in vitro, ex vivo [6]	photometry [6]	mouse, drosophila,	[7]
	GRAB-DA2h	DRD2	500/520 nm	280 % *	7 nM *	15-fold(NE)	50 ms *	7300 ms *	in vitro, ex vivo [6]	photometry [6]	mouse	[7]
	GRAB-rDA1m (red)	DRD2	565/595 nm	150 % *	95 nM *	15-fold(NE)	80 ms *<br>150 ms ****	770 ms *<br>5200 ms ****	in vitro, ex vivo [6]	photometry [6]	mouse, drosophila,	[7]
	GRAB-rDA1h (red)	DRD2	565/595 nm	100 % *	4 nM *	10-fold(NE)	60 ms *<br>80 ms ****	2150 ms *<br>11800 ms ****	in vitro, ex vivo [6]	photometry [6]	mouse	[7]
NE	nLight1.3	β2AR	490/516 nm	155 % *	760 nM *	50-fold(DA)	ND	ND	in vitro [1,4]	2-photon (aggregated signal) [8]	mouse	[1,8]
	GRAB-NE1m	α2AR	490/510 nm	230 % *	930 nM *	350-fold(DA)	72 ms *<br>37 ms ****	680 ms *<br>600 ms ****	in vitro, ex vivo [9]	photometry [9]	mouse, zebrafish	[9]
	GRAB-NE1h	α2AR	490/510 nm	130 % *	83 nM *	37-fold(DA)	36 ms *	1890 ms *	in vitro, ex vivo [9]	ND	zebrafish	[9]
5-HT	sLight1.3	5HT2A	490/516 nm	80 % *	650 nM *	NA	ND	ND	in vitro [1]	ND	ND	[1], see [10]
	GRAB-5HT1.0	5HT2C	490/510 nm	250 % *	14 nM *	NA	200 ms *<br>150 ms ****	3100 ms *<br>7220 ms ****	in vitro, ex vivo [11]	2-photon (aggregated signal or non-cellular ROIs)	mouse, drosophila	[11] †
Ach	GAch2.0	M3R	490/510 nm	90 % *	2100 nM **	NA	280 ms *	760 ms *	in vitro, ex vivo [12]	photometry, 2-photon (cellular ROIs) in mice	mouse, drosophila	[12]
	GAch3.0	M3R	492/510 nm	280 % *	2200 nM **	NA	105 ms ****	3700 ms ****	in vitro, ex vivo [13]	photometry [14], 2-photon (non-cellular ROIs) [14], mesoscope (aggregated signal) [15]	mouse, drosophila	[14]
Ado	GRAB-Ado1.0	A2AR	490/510 nm	120 % *	60 nM **	NA	68 ms *	16000 ms *	in vitro, ex vivo [16]	photometry [17]	mouse	[16,17]
eCB	GRAB-eCB2.0	CB1R	490/510 nm	210% (2-AG) *<br>210% (AEA) *	7200 nM (2-AG) *<br>500 nM (AEA) *	NA	1600 ms *<br>(2-AG)	11200 ms<br>(2-AG) *	in vitro, ex vivo [16]	photometry, 2-photon (non-cellular ROIs) [18]	mouse	[18] †
GRP	grpLight1.3	GRPR (= BB2R)	490/516 nm	ND	355 nM *	NA	ND	ND	in vitro, ex vivo [19]	photometry [19]	mouse	[19] †

Supplementary Table 1: Main properties of currently available GPCR sensors for neuromodulators

α2AR, β2AR: alpha and beta-adrenergic receptors, A2AR: adenosine 2 receptor, Ach: acetylcholine, Ado: adenosine, dFF_{max}: maximal increase in fluorescence between ligand-free and ligand saturated states, BB2R: bombesin 2 receptor, CB1R: cannabinoid receptor type 1, DRD1/DRD2: dopamine 1 or 2 receptor, DA: dopamine, eCB: endocannabinoid, Emis: emission wavelength, Exc: excitation wavelength, GRP: gastrin-related peptide, GRPR: gastrin-related peptide receptor, NA: not applicable, ND: not determined, NE: noradrenaline, M3R: muscarinic 3 receptor, 5HT: serotonin, 5HT2A and 5HT2C: serotonin 2A and 2C receptors

* estimated in HEK-293 cells following bath or puff ligand application; ** estimated in cultured neurons following bath or puff ligand application; *** estimated in brain slices following 1 electrical pulse (0.5-ms); **** estimated in brain slices following 10 electrical pulses (1-ms each) at 100Hz during 100ms; † not peer-reviewed at the time of this publication.

References:

1. Patriarchi T, Cho JR, Merten K, Howe MW, Marley A, Xiong W-H, et al. Ultrafast neuronal imaging of dopamine dynamics with designed genetically encoded sensors. Science. 2018;360:eaat4422.
2. Robinson JE, Coughlin GM, Hori AM, Cho JR, Mackey ED, Turan Z, et al. Optical dopamine monitoring with dLight1 reveals mesolimbic phenotypes in a mouse model of neurofibromatosis type 1. ELife. 2019;8:e48983.
3. Mohebi A, Pettibone JR, Hamid AA, Wong J-MT, Vinson LT, Patriarchi T, et al. Dissociable dopamine dynamics for learning and motivation. Nature. 2019;570:65–70.
4. Patriarchi T, Mohebi A, Sun J, Marley A, Liang R, Dong C, et al. An expanded palette of dopamine sensors for multiplex imaging in vivo. Nature Methods. 2020:1–9.
5. Sun F, Zeng J, Jing M, Zhou J, Feng J, Owen SF, et al. A Genetically Encoded Fluorescent Sensor Enables Rapid and Specific Detection of Dopamine in Flies, Fish, and Mice. Cell. 2018;174:481-496.e19.
6. Sun F, Zhou J, Dai B, Qian T, Zeng J, Li X, et al. New and improved GRAB fluorescent sensors for monitoring dopaminergic activity *in vivo*. Neuroscience; 2020.
7. Sun F, Zhou J, Dai B, Qian T, Zeng J, Li X, et al. Next-generation GRAB sensors for monitoring dopaminergic activity in vivo. Nature Methods. 2020:1–11.
8. Oe Y, Wang X, Patriarchi T, Konno A, Ozawa K, Yahagi K, et al. Distinct temporal integration of noradrenaline signaling by astrocytic second messengers during vigilance. Nat Commun. 2020;11:471.
9. Feng J, Zhang C, Lischinsky JE, Jing M, Zhou J, Wang H, et al. A Genetically Encoded Fluorescent Sensor for Rapid and Specific In Vivo Detection of Norepinephrine. Neuron. 2019;102:745-761.e8.
10. Andreoni A, Davis CMO, Tian L. Measuring brain chemistry using genetically encoded fluorescent sensors. Current Opinion in Biomedical Engineering. 2019;12:59–67.
11. Wan J, Peng W, Li X, Qian T, Song K, Zeng J, et al. A genetically encoded GRAB sensor for measuring serotonin dynamics in vivo. BioRxiv. 2020:2020.02.24.962282.
12. Jing M, Zhang P, Wang G, Feng J, Mesik L, Zeng J, et al. A genetically encoded fluorescent acetylcholine indicator for in vitro and in vivo studies. Nat Biotechnol. 2018;36:726–737.
13. Jing M, Li Y, Zeng J, Huang P, Skirzewski M, Kljakic O, et al. An optimized acetylcholine sensor for monitoring *in vivo* cholinergic activity. Neuroscience; 2019.
14. Jing M, Li Y, Zeng J, Huang P, Skirzewski M, Kljakic O, et al. An optimized acetylcholine sensor for monitoring in vivo cholinergic activity. Nature Methods. 2020;17:1139–1146.
15. Lohani S, Moberly AH, Benisty H, Landa B, Jing M, Li Y, et al. Dual color mesoscopic imaging reveals spatiotemporally heterogeneous coordination of cholinergic and neocortical activity. BioRxiv. 2020:2020.12.09.418632.
16. Wu Z, Cui Y, Wang H, Song K, Yuan Z, Dong A, et al. A GRAB sensor reveals activity-dependent non-vesicular somatodendritic adenosine release. BioRxiv. 2020:2020.05.04.075564.
17. Peng W, Wu Z, Song K, Zhang S, Li Y, Xu M. Regulation of sleep homeostasis mediator adenosine by basal forebrain glutamatergic neurons. Science. 2020;369.
18. Dong A, He K, Dudok B, Farrell JS, Guan W, Liput DJ, et al. A fluorescent sensor for spatiotemporally resolved endocannabinoid dynamics in vitro and in vivo. BioRxiv. 2020:2020.10.08.329169.
19. Melzer S, Newmark E, Mizuno GO, Hyun M, Philson AC, Quiroli E, et al. Bombesin-like peptide recruits disinhibitory cortical circuits and enhances fear memories. BioRxiv. 2020:2020.10.26.355123.
